# Sensory input is required for callosal axon targeting in the somatosensory cortex

**DOI:** 10.1186/1756-6606-6-53

**Published:** 2013-12-05

**Authors:** Ying Huang, Ning-Ning Song, Wei Lan, Qiong Zhang, Ling Zhang, Lei Zhang, Ling Hu, Jia-Yin Chen, Chun-Jie Zhao, Lingjiang Li, Lin Xu, Yu-Qiang Ding

**Affiliations:** 1 Key Laboratory of Arrhythmias, Ministry of Education, East Hospitial, Tongji University School of Medicine, 1239 Siping Road, Shanghai, 200092, China; 2 Department of Anatomy and Neurobiology, Tongji University School of Medicine, 1239 Siping Road, Shanghai, 200092, China; 3 Key Laboratory of Developmental Genes and Human Diseases, Ministry of Education, School of Medicine, Southeast University, 87 Dingjiaqiao Road, Nanjing, Jiangsu, 210009, China; 4 Institute of Mental Health, Second Xiangya Hospital of Central South University, Changsha, 410011, China; 5 Key Laboratory of Animal Models and Human Disease Mechanisms, Chinese Academy of Sciences and Yunnan Province, Kunming Institute of Zoology, Kunming, 650223, China

**Keywords:** Callosal projection, Sensory input, Axon pathfinding, Somatosensory cortex

## Abstract

**Background:**

Sensory input is generally thought to be necessary for refining and consolidating neuronal connections during brain development. We here report that cortical callosal axons in somatosensory cortex require sensory input for their target selection in contralateral cortex.

**Results:**

Eliminating sensory input to either hemisphere by unilateral transection of infraorbital nerve (ION) prevents target selection of callosal axons in contralateral cortex. Strikingly, blocking sensory input bilaterally, by simultaneously transecting both IONs, results in rescued callosal projection. In contrast, non-simultaneous bilateral ION transection has the same effect as unilateral transection. Similar results are obtained by lesion of whisker hair follicles. c-Fos-positive neurons in brain slices treated with KCl is decreased more in contralateral cortex with unilateral removal of sensory input, but decreased similarly in both cortices in mice with simultaneous bilateral removal of sensory input. Frequency of sEPSC of cortical neurons is also reduced in contralateral cortex with the unilateral removal of sensory input, but equally reduced on both sides with the bilateral removal of sensory input, suggesting that unbalanced bilateral sensory input might lead to mismatched neuronal activity between the two cortices and contribute to the formation of callosal projection.

**Conclusion:**

Our data demonstrate a critical role of balanced bilateral somatosensory input in the formation of callosal connections, and thus reveal a new role of sensory input in wiring brain circuits.

## Background

The corpus callosum is the largest commissural system in the mammalian brain and responsible for communication between the two cerebral hemispheres, which is highlighted by the findings from “split brain” patients with corpus callosotomy [1,2]. Callosal neurons are mainly located in layers II-III and V. Callosal axons cross the midline region and then find correct cortical areas in opposite hemisphere to establish callosal connection [3]. In mouse, the early-born layer V callosal neurons cross the midline before birth, while the late-born layer II-III callosal neurons cross the midline several days after birth [3-5].

Agenesis of the corpus callosum is a birth defect that occurs in over 50 different human congenital syndromes [3] and malformation of the corpus callosum is associated with mental retardation, cerebral palsy and schizophrenia [6-8]. Therefore, numerous studies have explored the molecular mechanism underlying the development of the corpus callosum. Evidence from mutant mice has shown that guidance molecules, such as Netrin [9] and Slit [10], transcription factors, such as Emx1 [11] and Pax6 [12], and intracellular molecules, such as P35 and Cdk5 [13], are involved in the midline crossing of callosal axons. However, how callosal axons select the correct target in the contralateral cortex is unclear.

Recent evidence shows that callosal neuron activity also plays an important role in the formation of their callosal connection. Expression of the inward rectifying potassium channel Kir2.1, which lowers the neuronal excitability, in callosal neurons in the somatosensory and visual cortices dramatically reduces callosal projections to the corresponding contralateral cortex [4,5]. After birth these two cortical areas undergo rapid development, such as formation of whisker-related barrel and topographic arrangement of ocular dominance, and these events are driven by continuous somatosensory and visual inputs, respectively [14-17]. These findings promote us to examine whether sensory input is required for the formation of callosal connection in the somatosensory cortex.

In this study, we found that unilateral transection of ION during early postnatal period arrested callosal projection to the contralateral cortex. Surprisingly, simultaneous bilateral transecting IONs did not obviously affect the callosal projection, whereas non-simultaneous bilateral transection also arrested the callosal projection. Similar results were obtained by lesion of whisker hair follicles. Our results not only show the requirement of sensory input in wiring of callosal connection, but also reveal the critical role of balanced bilateral sensory input in this developmental process.

## Results

### Unilateral transection of ION on either side abolishes callosal projection

To follow the outgrowth of callosal axons of somatosensory neurons, we delivered an EGFP expression construct into the pyramidal neurons of the somatosensory cortex by *in utero* electroporation at 15.5 dpc. Consistent with our previous finding [5], callosal axons of layer II-III cortical pyramidal neurons enter the white matter beneath the contralateral somatosensory cortex at postnatal day (P) 5, and initiate dense projections to the border region between the primary somatosensory cortex (S1) and secondary somatosensory cortex (S2) during P6-P9. After P9, this callosal projection did not change markedly but an increase in axon aborization in layers II-III and V was observed [4,5].

**Figure 1:**
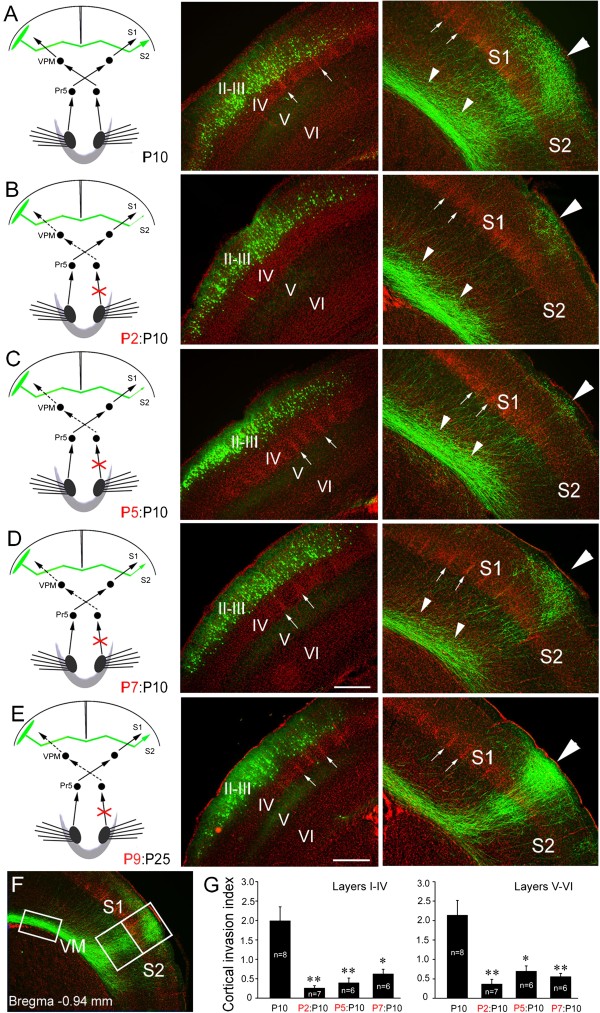
**Contralateral ION transections arrests target selection of layer II/III callosal neurons in the contralateral cortex.** Slices from P10 mouse brains counterstained with Hoechst (red) showing somatosensory callosal neurons expressing EGFP (green) in their cell bodies (middle panels) and axon terminals in the opposite hemisphere (right-hand panels). **(A)** Layer II/III callosal axons, labeled by *in utero* electroporation of an EGFP expression construct at 15.5 dpc, project extensively into the contralateral S1/S2 border cortex. **(B, C)** Projection to the cortex is arrested in the white matter beneath the S1/S2 border when afferent sensory input from the ION is excluded from P2 **(B)** and P5 **(C)**. **(D)** When ION is excluded from P7, more callosal axons enter the cortex by P10. **(E)** When ION is excluded from P9, however, callosal axons densely project to the border regions by P25. **(F)** Area of GFP immunofluorescence in the superficial (I-IV) and deep (V-VI) layers of S1/S2 border (large boxed area) and white matter (small boxed area) was measured, and the cortical invasion index was calculated as the area of axons in the cortex normalized to that in the white matter. **(G)** Cortical invasion index (see Methods); *P < 0.05, ** *P* < 0.01. Large triangles point to the S1/S2 border, small triangles point to callosal axons in the white matter beneath the S1/S2 border, and arrows point to boundary regions between two barrels. Pr5, principal sensory trigeminal nucleus; VPM, ventral posteriomedial thalamic nucleus; I-VI, cortical layers. Scale bars, 250 μm **(A-D)** and 300 μm **(E)**.

Thalamocortical circuits that relay somatosensory input from the orofacial region to the somatosensory cortices mature around P5 [16,17], and lowering neuronal excitability of the callosal neurons by expression of Kir2.1 dramatically reduces the callosal projections to the S1/S2 border region [5]. We wondered whether sensory input influences the timing of target zone invasion. The ION on one side carries the major orofacial somatosensory input to the contralateral somatosensory cortex [17], and we thus transected the ION at P2 on the side contralateral to the side of EGFP delivery in order to eliminate afferent sensory input to the callosal neurons. As shown in Figure 1A and B, callosal axons projected very densely to the S1/S2 border region in control brains, whereas after the ION transection few callosal axons were detected in the border region although a substantial number of callosal axons were situated in the white matter beneath the contralateral cortex (small triangles in Figure 1B). Transection of the ION at P2 leads to a failure of barrel formation in the S1 [16,17], and this may account for the targeting defect at the opposite S1/S2 border. Transection at P5, however, does not disrupt barrel formation [16,17], but would be expected to eliminate the later sensory input. Following transection of the contralateral ION at P5, callosal axons had also failed to invade the S1/S2 border region (Figure 1C, G), revealing that sensory input, but not barrel structures *per se*, is required for the callosal targeting in the contralateral cortex.

**Figure 2:**
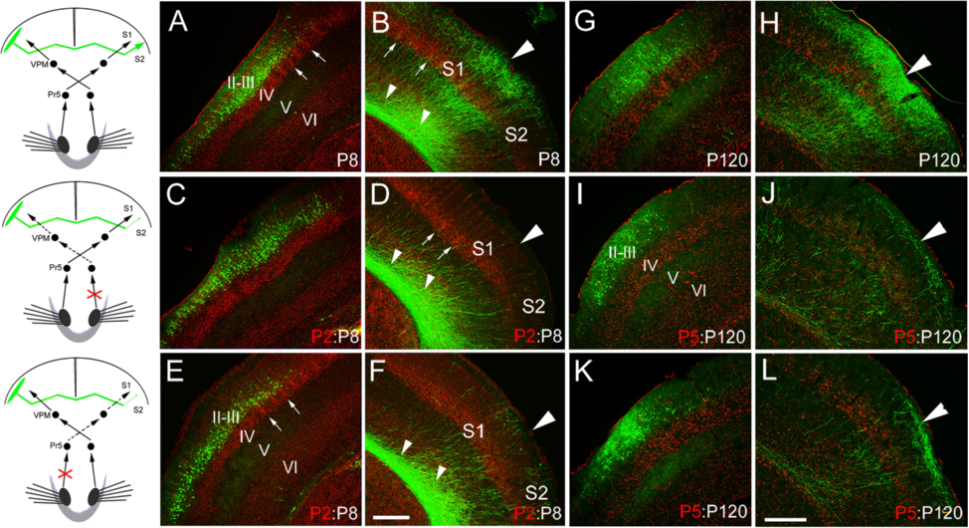
**Failure of callosal innervation is detected at P8, and still present in adulthood. (A-F)** Layer II/III somatosensory callosal neurons project densely to the S1/S2 border region of the contralateral somatosensory cortex at P8 in control brain **(A, B)**, but transection of the contralateral **(C, D)** or ipsilateral **(E, F)** ION at P2 greatly reduces callosal innervation of the S1/S2 border region at P8. **(G-L)** Dense callosal projection is present in control brain at P120 **(G, H)**, but callosal axons are dramatically reduced after P5 transection of the contralateral **(I, J)** or ipsilateral ION **(K, L)**. Large triangles point to the S1/S2 border **(A-L)**, small triangles point to callosal axons in the white matter beneath the S1/S2 border, and arrows point to boundary regions between two barrels **(A-F)**. For abbreviation, see Figure 1. Scale bars, 250 μm **(A-F)** and 350 μm **(G-L)**.

It is unlikely that the drastic reduction of callosal axons in the S1/S2 border cortex at P10 was due to the retraction of axons that have invaded the cortex at an earlier stage, because few axons were observed in the cortex at P8 after P2 (Figure 2C, D) and P5 ION transection. In addition, the reduction of callosal projection caused by P2 and P5 ION transection (Figure 2I, J) was also observed at P120, reflecting a permanent targeting defects. It should be noted that no ectopic callosal projections were found in the ipsilateral or contralateral cortex, showing that the failure of targeting S1/S2 border region is not caused by a switch of targeting site following the removal of sensory input. As development progressed, ION transection had progressively less effect. When ION transection was performed at P7, more axons were observed to have invaded the target cortex by P10 (Figure 1D, G). Sectioning the ION at P9 had no notable effect, when callosal targeting was examined at P25 (Figure 1E). Taken together, these data suggest that sensory input to callosal neurons is required continually for S1/S2 border cortex invasion until targeting is completed.

**Figure 3:**
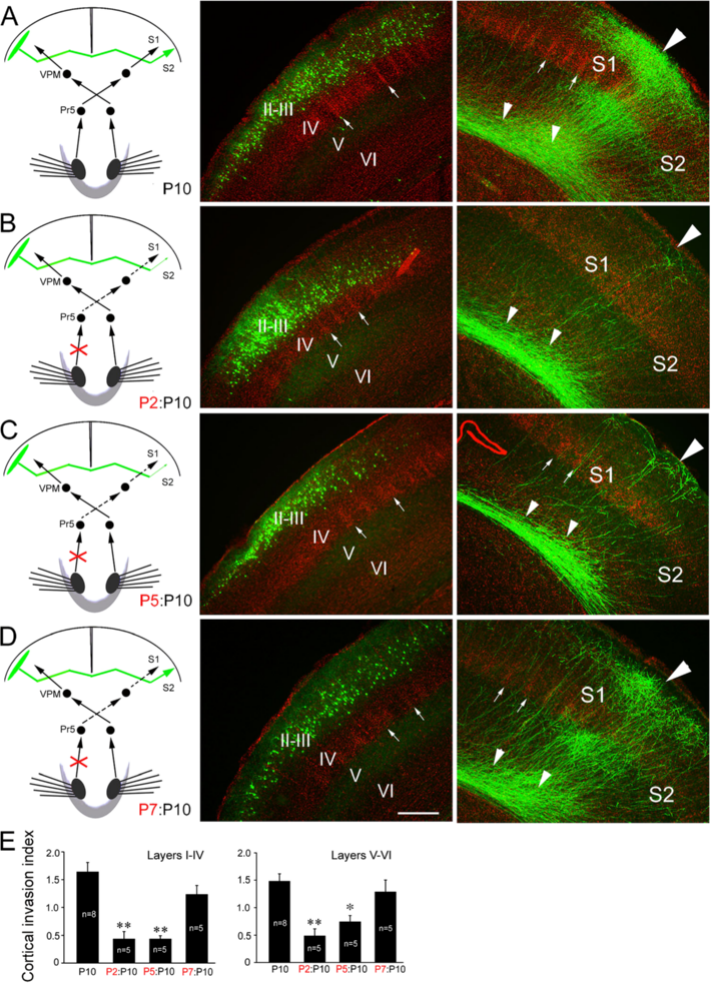
**Transection of ipsilateral ION also arrests target selection of layer II/III callosal neurons. (A)** Layer II/III callosal axons project extensively into the contralateral S1/S2 border cortex. **(B, C)** Transection of ION ipsilateral to callosal neurons at P2 **(B)** and P5 **(C)** arrests almost all callosal axon outgrowth into the S1/S2 border region. **(D)** Ipsilateral transection ION at P7, however, does not obviously affect callosal projections to the border regions. **(E)** Cortical invasion index (see Methods); *P < 0.05, **P < 0.01. Large triangles point to the S1/S2 border, small triangles point to callosal axons in the white matter beneath the S1/S2 border, and arrows point to boundary regions between two barrels. For abbreviation, see Figure 1. Scale bar, 250 μm.

We next asked whether sensory input to the targeted cortex is also required for the formation of this callosal projection. To this end, we transected the ION ipsilateral to the side of electroporation (Figure 3). Transection of the ipsilateral ION at P2 was also able to arrest callosal invasion of the contralateral S1/S2 border cortex (Figure 3B, E). This phenotype was likewise not due to barrel structure malformations in the targeting somatosensory cortex, because transection at P5 also precluded target innervation (Figure 3C, E). Ipsilateral ION transection at P7 or P9, however, had no notable effect examined at P10 (Figure 3D, E) or P21.

Similar to what were observed in the experiment of contralateral transection of ION, a drastic reduction of callosal axons in the S1/S2 border region at P10 was not due to the retraction of axons that have invaded the cortex at an earlier stage, because few axons were observed in the cortex at P8 after P2 (Figure 2E, F) and P5 ipsilateral ION transection. In addition, P2 and P5 ipsilateral ION transection also led to a permanent callosal projection defect, as shown by much fewer callosal axons in the border regions compared with control brains at P120 (Figure 2G, H, K, L). Thus, sensory input to the target cortex is also required for the formation of callosal connection in the somatosensory cortex.

### Callosal projection defect is rescued after simultaneous bilateral transection of IONs

**Figure 4:**
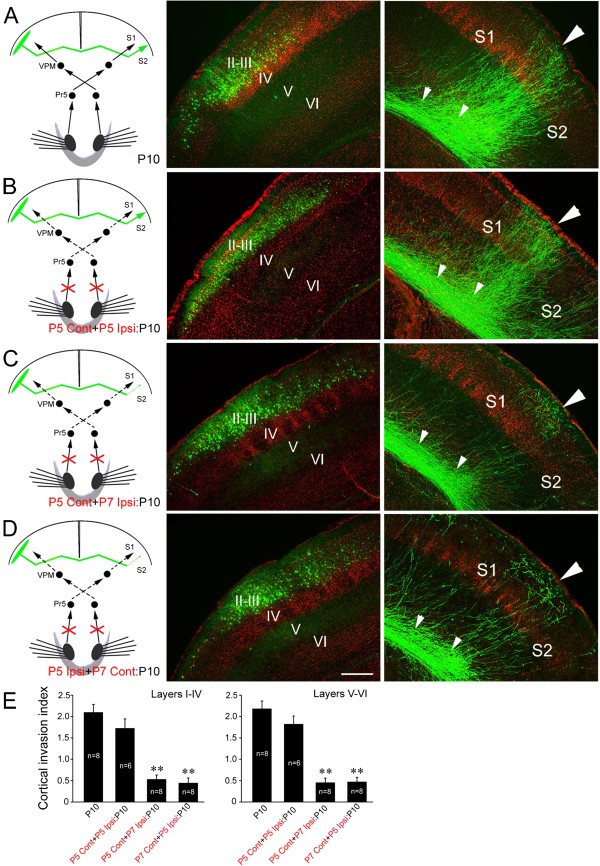
**Simultaneous bilateral ION transection has no effect but non-simultaneous bilateral transection blocks callosal target selection. (A)** Layer II/III callosal axons of control pups project densely to the contralateral S1/S2 border cortex, although axonal arborization is not evident under this artificial nutrition condition (compare with Figures 1A, 3A). **(B)** This pattern is maintained after bilateral ION transection at P5. **(C)** Transection of the contralateral ION at P5 and the ipsilateral ION at P7 abolishes callosal innervation of the S1/S2 border region. **(D)** A similar effect is observed after transecting the ipsilateral ION at P5 and contralateral ION at P7. **(E)** Cortical invasion index (see Methods); **P < 0.01. Large triangles point to the S1/S2 border, and small triangles point to callosal axons in the white matter beneath the S1/S2 border. For abbreviation, see Figure 1. Scale bar, 250 μm.

Having found that sensory input to both callosal neurons and their targeting cortex is required for callosal axon target selection, we were prompted to explore the effects of eliminating sensory input altogether. We transected both IONs simultaneously at P5 and examined the S1/S2 border projection at P10. Pups with bilateral ION transection could seldom survive for more than 2 days after the operation probably due to the inability of sucking enough milk. To prolong their survival until P10, they were gavage fed with milk. Under this nutrition condition, the dense S1/S2 border projection was still present in control pups although axonal arborization was not evident compared with that in control pups with normal nutrition (Figures 1A, 3A). To our surprise, the dense callosal projection to the S1/S2 border was also observed in pups with bilateral transection of IONs at P5 with no obvious difference compared with controls with intact sensory systems (Figure 4A, B, E), suggesting that balanced bilateral sensory input from the IONs, whether zero or non-zero, is critical for target selection by callosal somatosensory neurons. We pursued this further by assessing the effects of non-simultaneous bilateral ION transection. Transecting the contralateral ION at P5 and the ipsilateral ION at P7 (Figure 4C, E) or *vice versa* (Figure 4D, E) had equivalent effects, both procedures arrested the S1/S2 border projection similar to unilateral ION transection.

### Lesion of whisker hair follicles results in similar phenotypes

**Figure 5:**
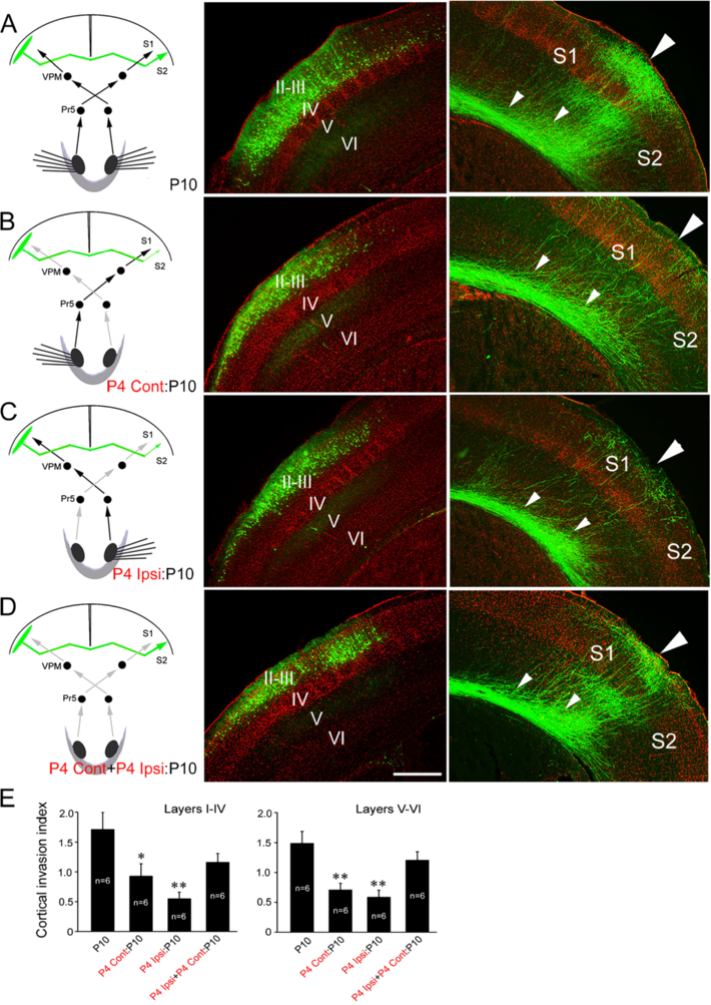
**Callosal projection is affected by unilateral but rescued by bilateral lesion of whisker hair follicles. (A)** Layer II/III callosal axons of control pups project densely to the contralateral S1/S2 border cortex. **(B, C)** Unilateral lesion of the hair follicles on either side at P4 also impairs callosal innervation of the S1/S2 border region. **(D)** Bilateral lesion of whisker hair follicles at P4 does not obviously affect the callosal projection. **(E)** Cortical invasion index (See Methods); *P < 0.05, **P < 0.01. Large triangles point to the S1/S2 border, and small triangles point to callosal axons in the white matter beneath the S1/S2 border. For abbreviation, see Figure 1. Scale bar, 250 μm.

To further confirm these results, we employed an alternative way to remove sensory input. Hair follicles of whiskers were destroyed by a heated thin wire at P4-P7 and callosal projections were examined at P10. Unilateral lesion of all whisker hair follicles on either side at P4 also led to a drastic reduction of callosal axons in the S1/S2 border region (Figure 5B, C, E), lesion at P5 was less effective and at P6-P7 had no effect. Like what were observed in bilateral ION transection at P5, callosal projection was largely normal when bilateral whisker hair follicles were destroyed at P4 (Figure 5D, E). By contrast, lesion of all whisker hair follicles on one side at P4 and the other side at P5 did not rescue the projection defect. Sensory input from the whiskers is the major component, but not all, of primary sensory information conducted by ION, and this may account for shorter time window for the lesion of whisker hair follicles in affecting callosal projection relative to that for ION transection. Nevertheless, these results further support the idea that balanced bilateral sensory input is required for the formation of callosal projection.

### Neuronal activity is altered in the somatosensory cortex after removal of sensory input

**Figure 6:**
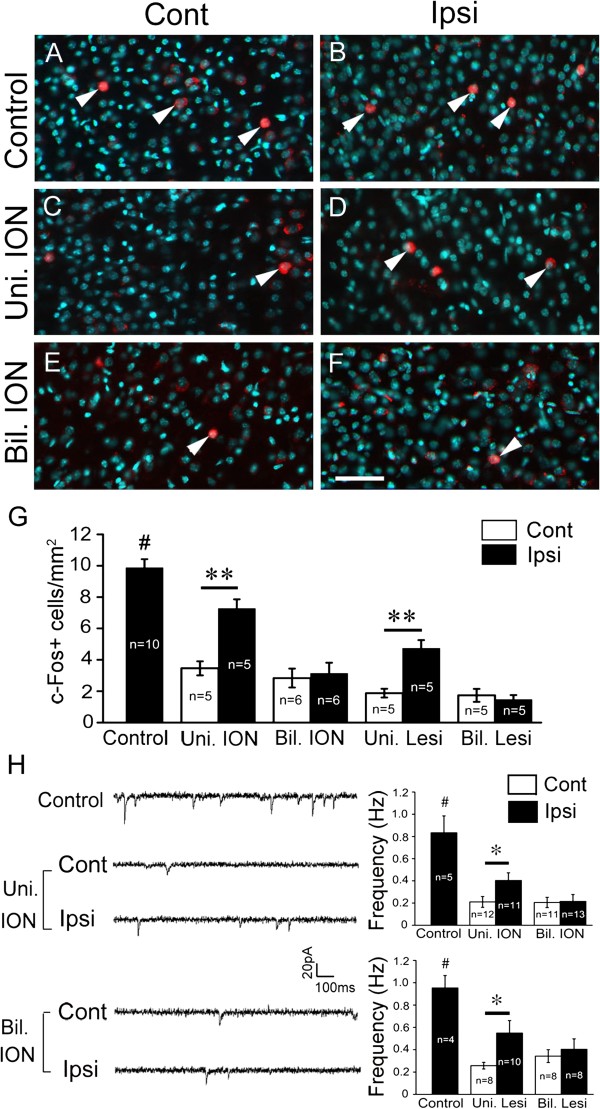
**Neuronal activity is altered in the somatosensory cortex after removal of sensory input. (A-G)** c-Fos expression was examined in P9 brain slices. Compared with control brain **(A, B)**, c-Fos-positive neurons are decreased more in number in P9 brain slices from the contralateral side compared with ipsilateral side **(C, D)** after P5 unilateral ION transection. While in the brain with P5 bilateral ION transection, c-Fos-positive neurons are similarly reduced in number on both sides **(E, F)**. Nuclear Hoechst counterstain is shown with pseudocolor (cyan), and c-Fos-positive neurons are indicated by triangles. Comparison of numbers of c-Fos-positive neurons is shown in **(G)**; animal numbers examined are indicated. **P < 0.01; #*P* < 0.01 (control vs the others). **(H)** Patch-clamp recording was performed in layer II/III neurons of the S1 region of brain slices prepared from P7 mice. Frequency of sEPSC is reduced on both sides in mice with bilateral unilateral removal of sensory input compared with control mice, but it shows more reduction in the contralateral side compared with that in the ipsilateral side. In mice with bilateral removal of sensory input, frequency of sEPSC of the cortical neurons in the S1 is equally reduced on both sides. Representative recordings in brain slice prepared from mice with ION transection are shown in left panel, and quantification of the sEPSC frequency is shown in right two panels. At least three mice were examined in each group, and neuron numbers for each recoding are indicated. *P < 0.05, #*P* < 0.01 (control vs the others). Bil. ION, bilateral transection of IONs; Bil. Lesi, bilateral lesion of whisker hair follicles; Cont, contralateral side; Ipsi, ipsilateral side; Uni. ION, unilateral transection of ION; Uni. Lesi, unilateral lesion of whisker hair follicles. Scale bar, 60 μm.

Unilateral or non-simultaneous bilateral removal of sensory input may disrupt the balance of bilateral sensory input to the two somatosensory cortices, which may in turn lead to mismatched activity between the two cortices. In contrast, neuron activity of the two cortices may match well after the simultaneous bilateral ION transection, although it may be reduced. To test this possibility, c-Fos was used to examine cortical neuronal activity in P9 brain slices prepared from mice with P5 unilateral or simultaneous bilateral removal of sensory input. It is well known that neurons can be excited by high concentration of potassium *in vitro*. Brain slices were incubated with 50 mM KCl for 10 min and c-Fos staining were performed 40 min later. In brain slices prepared from mice with unilateral removal of sensory input (transection of ION or lesion of whisker hair follicles on one side), c-Fos-positive neurons were decreased more in number in the contralateral brain slices compared with the ipsilateral slices (Figure 6A-D, G). In brain slices with simultaneous bilateral removal of sensory input, the number of c-Fos-positive neurons on the two sides was similarly decreased (Figure 6E-G). These results suggest that unilateral removal sensory input might lead to imbalanced neuronal activity between the two-side cortices, while simultaneous bilateral removal might not do so.

We next used a whole cell patch clamp technique to record the resting membrane potential, threshold for evoked action potential, and spontaneous excitatory postsynaptic current (sEPSC) of layer II/III cortical neuron in P7 mice with P5 unilateral or P5 bilateral ION transection. Compared to control mice, resting membrane potential and threshold for evoked action potential showed no difference in the two cortices of the mice with unilateral or bilateral ION transection, suggesting that electrical property of cortical neurons is not changed after P5 ION transection. However, frequency of sEPSC decreased differentially in the two side cortices with unilateral ION transection, as shown by more reduction on the side contralateral to ION transection compared to that of the ipsilateral side (Figure 6H). In contrast, in the mice with P5 bilateral ION transection, frequency of sEPSC was reduced on both sides with no statistical difference (Figure 6H). Similar results were obtained in mice with unilateral or bilateral lesion of whisker hair follicles (Figure 6H). Amplitude of sEPSC was not changed in unilateral or bilateral ION-transected mice relative to controls. Considering the critical role of sensory input in the maturation of somatosensory cortex, it might be possible that the reduced frequency of sEPSC may reflect less presynaptic glutamate release driven by peripheral sensory input, and imbalanced bilateral sensory input might lead to mismatched activity between the two-side cortices whereby contributing to defective callosal targeting in the contralateral cortex.

## Discussion

In this study, we removed somatosensory input by transecting the ION or lesion of whisker hair follicles to examine the effects of sensory input on the development of the corpus callosum. Our results demonstrate that synchronous bilateral sensory input is required for target selection of callosal neurons at system level. Unilateral transection of ION on either side at P2-P5 arrested the callosal projection, but it was less effective at P7 and showed no effect after P7 (Figures 1, 3). These results suggest that the establishment of callosal projection requires peripheral sensory input, and, like the role of sensory input in barrel formation in the S1 [16,17], there is also a “critical period” during which sensory input is required but no longer required once the projection is formed.

A previous study has shown that unilateral transection of ION at birth in rat disrupts the callosal projection in the S1/S2 border region when examined at postnatal one month [18]. Our data confirmed this finding in mouse, and further showed that the drastic reduction of callosal projection to the S1/S2 border region is due to the failure of callosal invasion of the S1/S2 cortex (Figures 1, 2 and 3). The most interesting finding is that bilateral transection of IONs at P5 did not obviously affect the callosal projection, whereas bilateral transection of IONs in the non-simultaneous way also arrested the projection (Figure 4). In addition to removal of peripheral sensory input, transection of IONs may also lead to other unknown effects that may contribute to the callosal projection phenotypes. To exclude this possibility, we employed an alternative method (lesion of whisker hair follicles) to remove sensory input, and obtained similar results (Figure 5). Thus, when bilateral sensory inputs are removed in the same spatial and temporal fashion, callosal projection is maintained. On the other hand, in the cases of unilateral or bilateral removal of sensory input in a non-simultaneous way, the balance of bilateral sensory input could be disrupted, and callosal invasion of target cortex could not progress thus leading to the drastic reduction of callosal projections. In support of this, neuronal activity of two cortices shown by c-Fos immunostaining is matched in the mice with simultaneous bilateral removal of sensory input although it is reduced on both sides, but not in those with unilateral removal of sensory input (Figure 6). Considering the important role of sensory input in the maturation of somatosensory cortex [16,17] and the different reduction of sEPSC frequency of cortical neurons (Figure 6), we speculate that peripheral sensory input may be a major factor for this mismatched neuronal activity between the two cortices in mice with unilateral removal of sensory input. However, it should be noted that our neurophysiological results are not sufficient to establish a causal link between the mismatched neuronal activity and defective callosal projection, and further studies are needed to explore this question.

Interestingly, unilateral and bilateral disruption of visual sensory input with monocular or binocular vision by enucleation, or dark rearing at birth does not prevent targeting of cortical callosal projections from innervating the contralateral cortex [19]. The essential role of balanced bilateral sensory input might explain this difference. Unlike the somatosensory system, visual input from one eye is delivered to both visual cortices, and thus either monocular or binocular enucleation or dark rearing would not disrupt the balanced sensory inputs in the two hemispheres. In contrast, unilateral lesion of the optic tract, which eliminates all visual input to one hemisphere, results in a nearly complete absence of callosal connections in the border region between the primary and secondary visual cortices [19]. Thus, it might be possible that callosal neurons in both somatosensory and visual cortices require matched bilateral sensory input to form their callosal connections.

It is well accepted that prior to initial contact formation, intrinsic developmental programs and external guidance cues are responsible for guiding growing axons into their target field [20-23], and sensory input is required for refining and consolidating neuronal connections in developing neuronal networks [24,25]. On the other hand, accumulated evidence indicates that neuronal activity is critical in axon pathfinding. For example, spontaneous rhythmic activity in early chick spinal cord influences distinct motor axon pathfinding decisions [26], and intraventricular injection of tetrodotoxin blocks cortical target selection of thalamocortical axons [27]. On the basis of these findings, it is likely that during early postnatal development sensory input-driven neuronal activity plays an important role in guiding growing axons to select correct target to establish proper neuronal connections.

## Conclusions

In this study, we found that the callosal projection to the contralateral cortex was arrested by unilateral transection of ION or lesion of all whisker follicles before P7. While simultaneous bilateral ION transection or lesion of whisker hair follicles did not obviously affect the callosal projection, whereas non-simultaneous bilateral transection of IONs or lesions whisker hair follicles arrested the callosal projection. Besides, the neuronal activity is altered in the somatosensory cortex after removal of sensory input. Our results not only show the requirement of sensory input in wiring of callosal connection, but also reveal the critical role of balanced bilateral sensory input in this developmental process.

## Materials and methods

### In utero electroporation

*In utero* electroporation was performed on timed pregnant C57BL/6 mice at 15.5 days post-coitum (dpc) as previously described [5]. After anesthesia with sodium pentobarbital, pregnant mice were subjected to abdominal incision to expose the uterus. The CAG-EGFP plasmid (1.5 μg/μl) was injected into the lateral ventricle with a glass capillary through the uterine wall. Electric pulses were then delivered to the embryos by gently clasping their heads with forceps-shaped electrodes connected to a square-pulse generator, ECM-830 (BTX; Holliston, MA). The pups of either sex were allowed to survive to different postnatal stages, and perfused with 4% of paraformaldehyde in 0.01 M phosphate buffered saline (pH 7.4) under deep anesthesia. Animal care and experimental protocols were approved by the Animal Center of Tongji University School of Medicine, China.

### ION transection

The IONs of P2-P9 pups were severed as described previously [16,17]. Since pups with bilateral IONs transection were unable to suck breast milk, they were gavage fed with milk 3-5 times daily, and control pups with sham operation in this set of experiment were treated in the same way.

### Electrophysiological recording

To examine electrophysiological changes of cortical neuron after unilateral or bilateral removal of sensory input, patch-clump recording was performed in brain slice. Brains from P7 mice of either sex were removed and transverse slices (350 μm) were cut on a vibrating microslicer (Leica VT1200, Germany). Whole-cell patch-clamp recordings were performed after the brain slices at a proximate level of Bregma -0.22 – -1.94 mm were incubated for 1 h in external artificial CSF (in mM: NaCl 117, KCl 3.6, CaCl_2_ 2.5, MgCl_2_ 1.2, NaH_2_PO_4_ 1.2, NaHCO_3_ 25 and glucose 11) which was bubbled continuously with carbogen (95%O_2_/5%CO_2_). Recording pipettes with resistances of 3–5 MΩ were pulled from borosilicate glass (P-97; Sutter Instruments, Novato, CA) and filled with a solution of (in mM) potassium gluconate 135, KCl 5, CaCl_2_ 0.5, MgCl_2_ 2, EGTA 5, HEPES 5 and ATP-Mg 5. Resting membrane potential and the threshold for evoking action potential were measured in layer II-III cortical neurons in current clamp mode, and sEPSC (spontaneous excitatory post synaptic current) was also recorded in voltage clamp mode held at -70 mV. The signals were amplified with an Axopatch 700B amplifier (Molecular Devices, Sunnyvale, CA), filtered at 2 kHz, and digitized at 5 kHz. Four-thirteen neurons were recorded in each group, and data were stored with a personal computer using software of pCLAMP 10 and analyzed with Mini Analysis (Synaptosoft Inc., Fort Lee, NJ). Comparisons were performed using the One-Way ANOVA with *post-hoc* Fisher’s LSD test, and *P* values less than 0.05 were considered statistically significant.

### Immunohistochemistry

Fixed brains were cut into 40 μm-thick transverse sections and processed for GFP (1:2000; Invitrogen, Grand Island, NY) immunostaining as described in our previous study [5]. Sections were counterstained with Hoechst 33258 (Sigma, St. Louis, MO) to clearly identify the morphological features defining somatosensory cortex.

To examine the changes of neuronal activity in the somatosensory cortex after unilateral or bilateral removal of sensory input, c-Fos immunostaining was performed in P9 brain slice (400 μm) prepared as mentioned above. After being incubated in the oxygenated artificial CSF for 1 h, brain slices were incubated in 50 mM KCl (Sigma) in CSF for 10 min and then with CSF without KCl for 40 min. After fixation, brain slices were cut into 20 μm-thick sections using a cryostat and processed for c-Fos immunostaining (1:1000; Santa Cruz, Dallas, TX). Immunostained sections were counterstained with Hoechst (Sigma), and c-Fos-positive neurons in the S1/S2 border region were counted in all sections obtained. Comparisons were performed using the One-Way ANOVA with *post-hoc* Fisher’s LSD test and *P* values less than 0.05 were considered statistically significant.

### Image analysis

Medio-lateral extents of GFP-labeled neurons in electroporated cortices varied, and brains containing GFP^+^ neurons located within the S1-S2 region were included. Every sixth sections were collected as one set section, in which about three sections were included at the approximate level of Bregma -0.22 – -1.94 mm in most brain samples [28]. These three sections were used for image analysis because callosal projection shows high consistency at this level along the rostro-caudal axis in control brains. Callosal axons projects primarily to the S1/S2 border region [5], and we thus quantified callosal axons in this area on the contralateral side. As shown in Figure 1F, ImageJ software (NIH) was used to measure the area of GFP immunofluorescence in the superficial (I-IV) and deep (V-VI) layers of the S1/S2 border region; this area covered the entire S1/S2 border region target territory. The cortical invasion index was calculated as the area of axons in the cortex normalized to that in the white matter (a small boxed area in Figure 1F). Individual data were pooled into groups and compared by One-Way ANOVA with *post-hoc* Fisher’s LSD test for multiple comparisons versus the control.

## Abbreviations

Bil. ION: Bilateral transection of IONs; Bil. Lesi: Bilateral lesion of whisker hair follicles; Cont: Contralateral side; dpc: Days post-coitum; ION: Infraorbital nerve; Ipsi: Ipsilateral side; P5: Postnatal day 5; Pr5: Principal sensory trigeminal nucleus; S1: Primary somatosensory cortex; S2: Secondary somatosensory cortex; sEPSC: Spontaneous excitatory postsynaptic current; VPM: Ventral posteriomedial thalamic nucleus; Uni. ION: Unilateral transection of ION; Uni. Lesi: Unilateral lesion of whisker hair follicles.

## Competing interests

The authors declare that they have no competing interests.

## Authors’ contribution

YQD conceived the study. YH, NNS, CJZ, LJL, LX and YQD designed the research. YH, NNS, WL, QZ, LeiZ, LH and JYC performed immunostaining and analyzed the data. LingZ performed electrophysiology and analyzed its data. LeiZ performed *in utero* electroporation. YH, NNS and YQD wrote the paper. All authors read and approved the final manuscript.
